# The Versatile Applications of Calix[4]resorcinarene-Based Cavitands

**DOI:** 10.3390/molecules29245854

**Published:** 2024-12-11

**Authors:** Kaiya Wang, Kejia Yan, Qian Liu, Zhiyao Wang, Xiao-Yu Hu

**Affiliations:** 1 College of Materials Science and Technology, Nanjing University of Aeronautics and Astronautics, Nanjing 211106, China; yankejia@nuaa.edu.cn (K.Y.); liuqian@nuaa.edu.cn (Q.L.); histtatp@nuaa.edu.cn (Z.W.); huxy@nuaa.edu.cn (X.-Y.H.); 2 College of Chemistry and Materials, Jiangxi Normal University, Nanchang 330022, China

**Keywords:** cavitands, molecular recognition, catalysis, supramolecular chemistry

## Abstract

The advancement of synthetic host–guest chemistry has played a pivotal role in exploring and quantifying weak non-covalent interactions, unraveling the intricacies of molecular recognition in both chemical and biological systems. Macrocycles, particularly calix[4]resorcinarene-based cavitands, have demonstrated significant utility in receptor design, facilitating the creation of intricately organized architectures. Within the realm of macrocycles, these cavitands stand out as privileged scaffolds owing to their synthetic adaptability, excellent topological structures, and unique recognition properties. So far, extensive investigations have been conducted on various applications of calix[4]resorcinarene-based cavitands. In this review, we will elaborate on their diverse functions, including catalysis, separation and purification, polymeric materials, sensing, battery materials, as well as drug delivery. This review aims to provide a holistic understanding of the multifaceted roles of calix[4]resorcinarene-based cavitands across various applications, shedding light on their contributions to advancing the field of supramolecular chemistry.

## 1. Introduction

Molecular recognition is a critical issue in the study of supramolecular chemistry and of great significance in the development of life. Inspired by nature, biologists originally proposed the “lock–key” model to explain the specificity of enzyme-substrate binding [1,2,3]. The ubiquitous occurrence of macrocycles in molecular recognition studies was inspired by natural receptors, helping to decipher phenomena observed in their biological equivalents. The exploitation of a structurally novel and functionally specific macrocycle host has always been a hot topic in supramolecular chemistry and a target for supramolecular chemistry researchers to pursue relentlessly. With the development of supramolecular chemistry, a variety of hosts have been synthesized and successfully applied to enzyme mimicry. Macrocyclic compounds, such as crown ethers, cyclodextrins, calixarenes, and pillar-arenes, are known to be supramolecular hosts that can accommodate a variety of guest molecules into their cavities to form host–guest complexes [4,5,6,7,8,9]. Calix[4]resorcin-arenes, as a special category of calixarene, are normally synthesized in high yields by condensation reaction between resorcinol and aldehyde [10,11,12,13]. Their distinctive three-dimensional structures offer almost unlimited derivation abilities through modification at their upper rim, lower rim, and OH groups [14,15,16]. Consequently, they have demonstrated remarkable versatility as fundamental building blocks in the field of supramolecular chemistry. Cavitands are prepared from resorcin-arenes by bridging their adjacent phenolic hydroxy groups to modulate the degree of conformational rigidity [17]. The resulting cavitand structure often resembles a molecular bowl or cup with a concave surface [18,19]. The empty cavity can selectively host or encapsulate smaller guest molecules through non-covalent interactions, such as hydrogen–halogen bonding [20,21,22,23], hydrophobic effect [24,25], cation–anion–π [26,27,28,29,30,31], and π–π stacking interactions [32,33,34]. Calix[4]resorcinarene-based cavitands have gained significant attention in supramolecular chemistry due to their remarkable capability for selective recognition and binding of specific guest molecules [35].

Cavitands have progressed through three intricately linked phases, revolving around synthesis, molecular recognition, and supramolecular assembly [10,36,37]. A comprehensive array of synthetic techniques has emerged for functionalizing cavitands to tailor their properties and elucidate the basic principles of host–guest chemistry [37,38]. In many instances, well-established synthetic methodologies were modified to accommodate the specific substrates, possessing complementary shape and size [39,40]. Cavitand chemistry, alongside various other synthetic macrocycles, played a crucial role in elucidating, evaluating, and harmonizing the mechanisms that are responsible for molecular recognition and assembly [41,42,43]. The most notable outcome is a set of guidelines for engineering more precise and accurate molecular-level interactions, which are essential for enhancing selectivity in recognition processes [44,45,46]. Following this initial surge in the above endeavors, the attention shifted towards investigating the application of cavitands. It is within this realm that researchers should direct their creativity to elevate the discipline to new heights [47,48,49]. We believe that now is the opportune time to summarize the versatile applications of cavitands.

In this review, we aim to provide a brief overview of the various applications realized so far based on calix[4]resorcinarene-based cavitands. The discussion will pivot towards catalysis, separation and purification, polymeric materials, sensing, battery materials, as well as drug delivery. The showcased examples in this review are not intended to be exhaustive, but rather serve as exemplars of the virtually boundless potential of cavitands in providing innovative solutions across a wide range of disciplines, spanning from biology to materials science, offering researchers a convenient avenue for accessing the most seminal works [50,51,52]. Examples from the last five years or so are discussed in this review, and readers interested in previous works are referred to in the above review. It is hoped that this review will provide a systematic and concise summary for researchers developing calix[4]resorcinarene-based applications.

## 2. Catalysis

**Figure 1:**
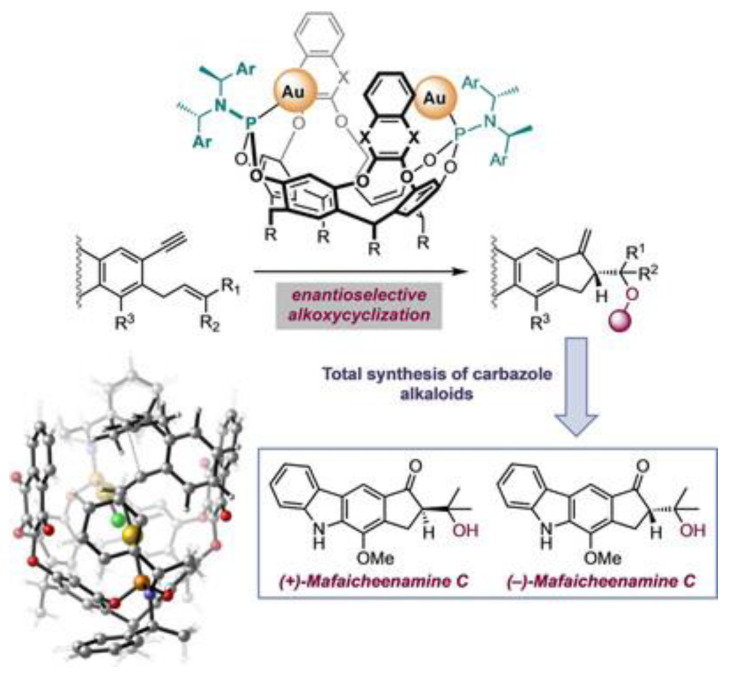
Schematic illustration of Gold(I)-cavitand catalysts for the enantioselective alkoxy-cyclization of 1,6-enynes. Reproduced from [61] with permission from the Angewandte Chemie International Edition.

Supramolecular catalysis is an important topic in supramolecular chemistry [53,54,55]. As a well-known supramolecular container, calix[4]resorcinarene-based cavitands have been extensively used as a molecular platform to build up various supramolecular catalysts [56,57,58]. While they exhibit notable success in influencing reaction kinetics and selectivity, challenges such as product inhibition remain, emphasizing the need for further exploration and refinement in the pursuit of realizing true catalytic processes within these confined molecular environments [59,60]. One of the primary approaches is to incorporate ligands for transition metal coordination. For instance, Echavarren et al. employed the gold-complexed cavitand for the enantioselective alkoxy-cyclization of 1,6-enynes with satisfactory yields (Figure 1) [61]. Gradually, there is a shift in trend towards employing larger-sized reactors assembled through complexation with various guests, rather than solely relying on the cavity as the nano-sized reactor.

**Figure 2:**
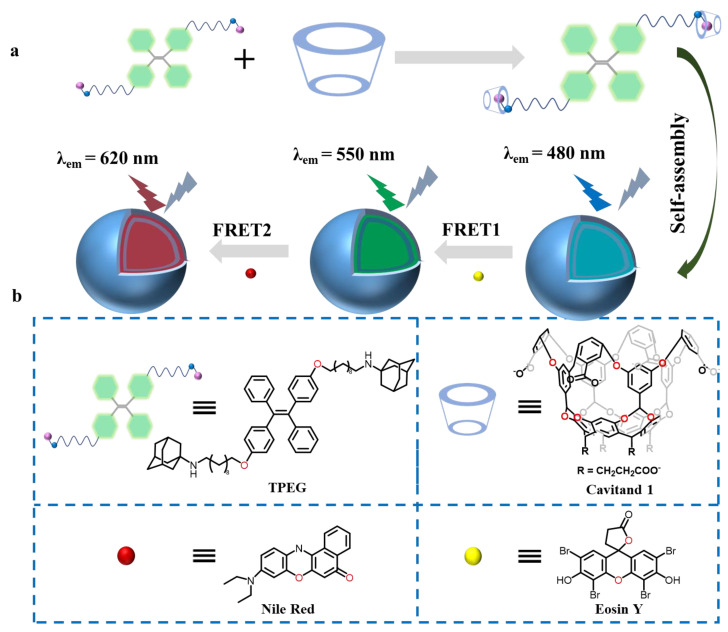
(**a**) Schematic illustration of a supramolecular artificial light-harvesting system with two-step sequential energy transfer in aqueous solution. (**b**) Chemical structures of the ALHSs. Reproduced from [62] with permission from Chemical communications.

To circumvent the drawbacks of the limited size of cavitand hosts, our group recently fabricated a novel artificial light-harvesting system (ALHS) with a sequential energy transfer process for efficient photocatalysis. This system was constructed from a water-soluble cavitand (**1**), a tetraphenyl-ethylene-functionalized di-amantadine derivative (**TPEG**), and fluorescent dyes Eosin Y (EsY) and Nile Red (NiR) (Figure 2) [62]. Cavitand (**1**) binds well with **TPEG** through a hydrophobic effect and further assembles into supramolecular nanoparticles **1⸧TPEG**, which exhibit a strong Tyndall effect. Due to the good overlap between the emission of **1⸧TPEG** and the absorption of EsY, the color of the **1⸧TPEG** solution changes from blue to green when ESY is added as the first receptor, indicating that the first step of the FRET (Forster Resonance Energy Transfer) procedure has taken place. Furthermore, the absorption of NiR overlapped well with the emission of **1⸧TPEG⸧ESY** and was chosen as the second receptor. When NIR was added, the **1⸧TPEG⸧ESY** solution changed from green to red, showing that the second step of the FRET process had occurred. To better mimic natural photosynthesis, the system was employed as catalyst for the photocatalysis of cross-dehydrogenative coupling (CDC) reaction in aqueous solution and exhibited excellent yields, which reached 80%.

**Figure 3:**
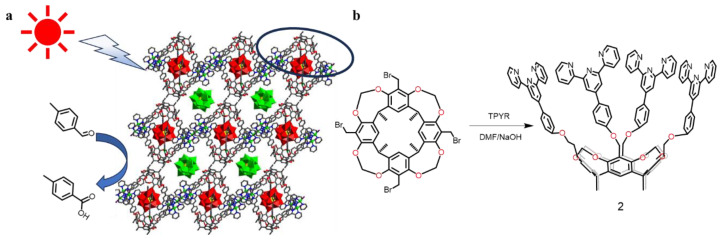
(**a**) Schematic illustration of a supramolecular artificial light-harvesting system. The circled part represents cavitand (**2**) (**b**) Synthetic scheme for cavitand (**2**). Reproduced from [63]. This is an open access article distributed under the Creative Commons Attribution License.

Similarly, Ma’s group has recently researched a novel calix[4] resorcinarene-based photocatalytic system (Figure 3) [63]. Cavitand (**2**) is modified by four terpyridines that can be coordinated with two copper(II) for the formation of a dimeric supramolecular nano-capsule through aromatic π–π stackings. The nano-capsules interacted with each other through encapsulated poly-oxometallate (POM) to form a photosensitizer, which resulted in a surprising visible light absorption effect. Under simulated solar light excitation, this system exhibits significantly increased photocatalytic activity for the oxidation of benzaldehyde. In order to investigate the photocatalytic activity of the system, suitable catalytic conditions were examined under different solvents, catalyst amounts and light conditions. The best reactivity was found in aqueous solution with 20 mg of catalyst under radiant light. In further detail, the system was performed for five cycles, and the reaction yields still reached 88.40%, indicating that the system retains good photocatalytic activity. The above two examples provide a novel strategy for utilizing cavitands in the field of supramolecular catalysis.

## 3. Separation and Purification

Natural systems showcase remarkable precision in the separation of molecules and ions, as evidenced by the functioning of potassium channels within the human body [64]. Notably, these channels selectively facilitate the permeation of potassium ions while effectively impeding smaller sodium ions, exemplifying a sophisticated mechanism for ion discrimination [65,66]. Cavitands can also be employed in extraction processes for selectively capturing and isolating specific molecules from mixtures, contributing to the purification of chemicals [67]. Incorporating supramolecular receptors into porous networks is a developing approach for purification of organic microcontaminants from water [68,69,70,71].

**Figure 4:**
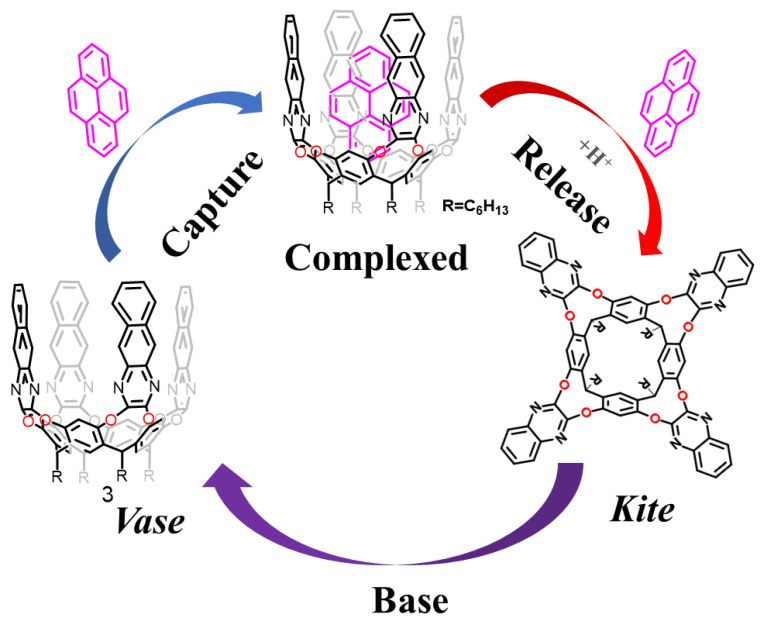
Schematic representation of pH-driven PAH uptake and release by cavitand (**3**).

Recently, Dalcanale et al. synthesized a cavitand receptor modified by four benzo-quinoxaline walls, which has a deeper hydrophobic cavity that can be used to capture larger PAHs (Polycyclic Aromatic Hydrocarbons) (Figure 4) [72]. Encapsulation of PAHs occurs in the vase form of cavitand (**3**), while release requires switching to the kite form. They embedded the cavitand receptor in an electro-spun polyacrylonitrile matrix to form a highly efficient, renewable membrane that was used to remove PAHs from water. Thermogravimetric analysis (TGA) of cavitand receptors at 400 °C revealed a less than 10% weight loss. Meanwhile, the fiber membrane was not damaged during sample desorption by gas chromatography at 270 °C. These two realizations are a strong indication of the excellent thermal stability of the cavitand. SPME–GC–MS analysis was used to evaluate the ability of the receptor to extract target PAHs from water samples. The results obtained showed a high level of affinity for all analytes studied. UV and fluorescence experiments in solution on the cavitand receptor revealed a unique phenomenon: the addition of TFA protonates the solution, transforming it from an open vase conformation to a closed kite conformation, which can burst some of the fluorescence emission. Treatment of the receptor material with an aqueous ammonia solution converts it to an open vase conformation, leading to recovery of fluorescence emission. This phenomenon demonstrates excellent filtration performance and pH-driven releasing ability.

**Figure 5:**
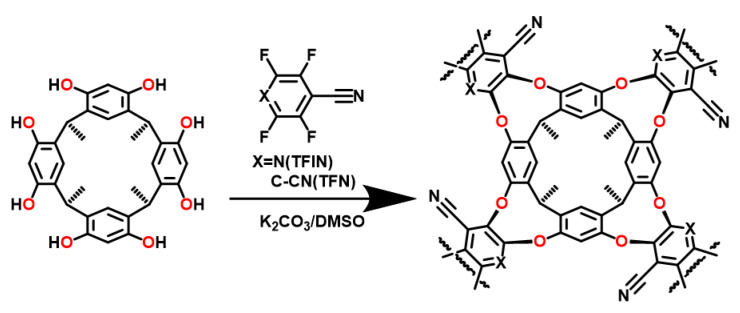
Synthetic scheme for separation material based on cavitand (**4**).

Similarly, Dichtel et al. have incorporated the calix[4]resorcinarene-based cavitand into a polymer adsorbent with a high surface area and porosity (Figure 5) [73]. The cavitand-based material (**4**) was synthesized by nucleophilic aromatic substitution of tetrafluoro-isonicotinonitrile (TFIN) or tetrafluoro-nitrilonitrile (TFN) with resorcinarene in anhydrous dimethyl-sulfoxide (DMSO) in the presence of K_2_CO_3_. The findings from the batch adsorption experiments suggest that **4** exhibits a better affinity for trihalomethanes in comparison to the activated carbon. TGA shows that, under appropriate conditions, the cavitand materials can be regenerated and reused many times without significant loss of performance. These excellent properties are achieved through a suitably designed structure. Remarkably, the cavitand materials outperform commercial resins and activated carbon in affinity for halomethane and 1,4-dioxane. These two materials exhibit potential for water treatment and underscore the significance of employing supramolecular receptors in the creation of adsorbents for remediation of pervasive and challenging-to-remove micropollutants.

**Figure 6:**
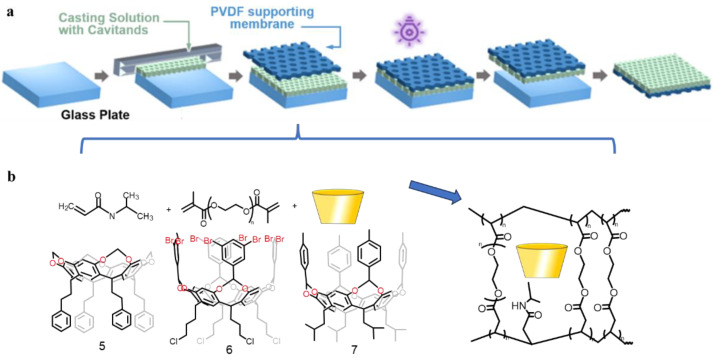
(**a**) The preparation of hydrogel composite membranes. (**b**) The synthetic process for the cavitand network and structural cavitand (**5**–**7**) network. Reproduced from [74] with permission from Separation and Purification Technology.

As well as addressing contaminants in mixtures, cavitands can also be employed to adsorb proteins of specific structures in order to accomplish the goal of protein isolation and purification. Protein separation is important in bioengineering and bioscience, especially in biotechnology, such as in pathogen identification, biosensor fabrication and protein crystallization. Recently, Jiang’s group constructed a hydrogel composite membrane (HCM) for efficient adsorption of bovine serum albumin (BSA), which introduces cavitand (**5**–**7**) with a porous structure and hydrophobicity into a hydrogel material based on polyethylene glycol diacrylate (PEGDA) and poly-N-isopropylacrylamide (PNIPAm) (Figure 6) [74]. Infrared spectra characterization information indicates that the hydrogel prepolymer undergoes photoinitiated polymerization to form a cross-linked network structure. Scanning electron microscopy (SEM) demonstrated that the surface of the hydrogel composite film was flat and dense, indicating excellent compatibility and tight bonding between the layers. Tensile tests demonstrated the hydrogel’s good mechanical strength. Molecular dynamics simulations verified that the role of the cavitand in hindering the diffusion of NIPAM molecules during polymerization is important to achieve the modulation of the HCM structure with polymer network properties from the interior to the surface of the material. Subsequently, adsorption experiments on bovine serum proteins with different hydrogel films revealed that the introduced cavitand play an important role as anion receptor during the protein adsorption process: the anion receptor makes an interfacial charge opposite to that of the proteins, and then immobilizes the proteins through electrostatic interactions. This offers a broad direction for scientists to develop protein separation materials with excellent properties in the future.

## 4. Polymeric Materials

Cavitands can be used to create supramolecular polymeric materials through self-assembly processes because of their unique construction [75,76,77]. This unique structural framework allows for the creation of materials with exceptional properties, opening up possibilities for diverse applications in fields such as optics and electronics. The significance of these advancements becomes particularly pronounced in the realm of nanotechnology, where the creation of well-defined nanostructures is a paramount objective [78]. Numerous studies have exemplified the potential of cavitands in crafting supramolecular polymeric materials with tailored functionalities.

**Figure 7:**
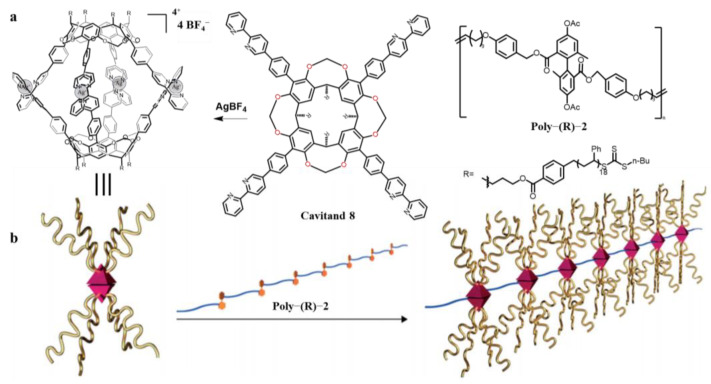
(**a**) Synthetic scheme of the graft copolymer derived from cavitand (**8**). (**b**) Schematic representation of the polymerisation of the cavitand (**8**). Reproduced from [79] with permission from the Angewandte Chemie International Edition.

For instance, Haino et al. constructed unique self-healing graft copolymer materials by means of supramolecular chemistry based on cavitand (**8**) [79] (Figure 7). Graft copolymers are a member of the block copolymer family, which consists of a main chain and multiple side chains. They designed cavitand-based self-assembling ligand capsules to bind to multiple guest sites on polyesters to form a novel supramolecular graft polymer. Atomic Force Microscopy (AFM) images demonstrate that the advanced and uniform polymer networks are the result of the formation of grafted structures through host–guest complexation. Differential scanning calorimetry (DSC) studies showed that the introduction of capsule side chains resulted in a uniform temperature change in glass transition temperature. The prepared supramolecular gel material was prepared for several tensile cutting experiments, showing good self-healing properties. This research proposes new methods for fabricating supramolecular polymeric materials that exhibit properties and functions similar to conventional polymer materials.

**Figure 8:**
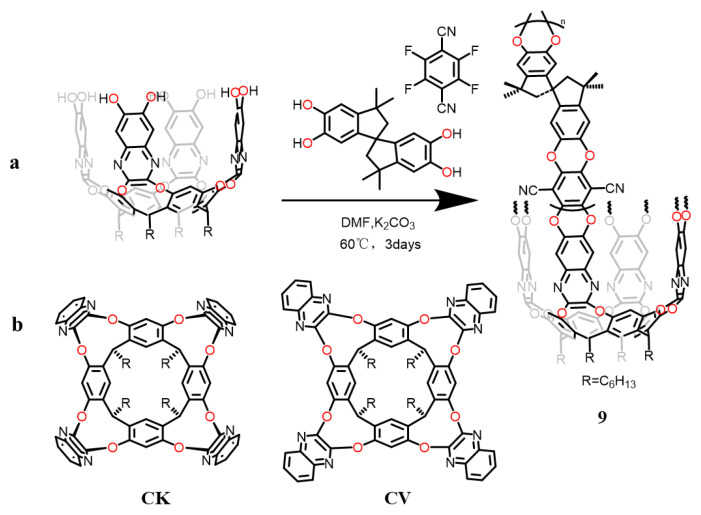
(**a**) Synthesis of cavitand (**9**). (**b**) Structure of kite cavitand and vase cavitand.

Auxetics, consisting of a distinctive material exhibiting the Negative Poisson’s Ratio (NPR) effect, have garnered significant attention owing to their remarkable propensity for lateral deformation, setting them apart from conventional materials. Dalcanale’s team developed a molecular stretching polymer by integrating conformationally expandable calix[4]resorcinarene-based cavitand (**9**) as crosslinker in a rigid polymer with intrinsic microporosity (PIM) (Figure 8) [80]. Incorporation of PIM to cavitand **9**, which can transform from a compact vase cavitand (**CV**) into kite cavitand (**CK**) form under mechanical stress, supplied the necessary kinematic response to the NPR at the macro level. The material’s outstandingly reversible pull-up behaviour was confirmed through mechanical testing of films created by blending cavitand **9** with virgin PIM utilizing digital image correlation techniques. Cavitands lacking hydroxyl groups at the upper edges were used for control experiments, and no conformational changes were observed, suggesting that covalent cross-linking of the quinoline walls for the induction of conformational transition and embedding the methyl groups in the upper end of the cavitand to block conformational changes are necessary. The experimental analysis showed no deformation behavior, indicating that the change in cavity conformation is an important cause of deformation. The observation of these two control experiments reveals that the NPR nature of materials arises solely from conformational changes induced by the cavitands.

## 5. Sensing

**Figure 9:**
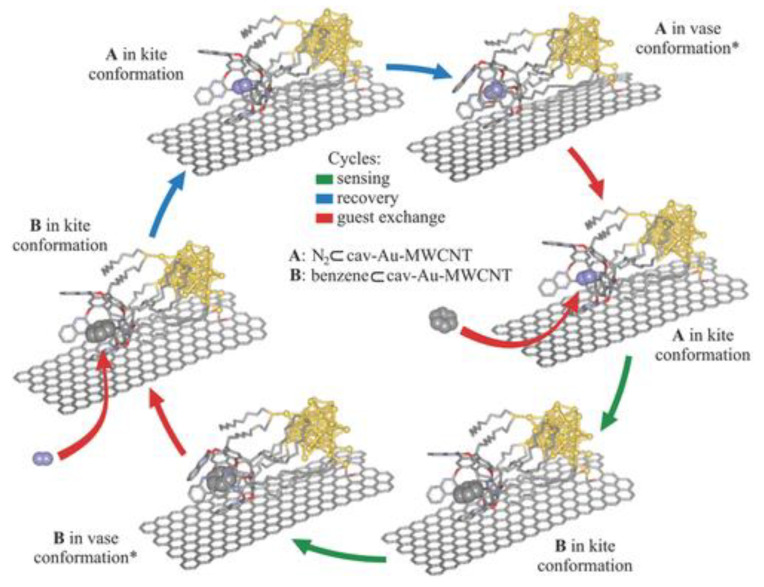
Schematic representation of benzene sensing and recovery mechanism. Benzene and nitrogen molecules are shown as CPK models. Hydrogen atoms are omitted for clarity. Notes: *crucial structures in the sensing process. Reproduced from [87] with permission from Advanced Functional Materials.

Cavitands can also be modified with different functional groups to be functionalized in order to selectively bind to specific guest molecules, making them useful as components in sensor devices for detecting analytes in environmental, biological, or chemical samples [81,82]. Numerous reports have been published in which cavitand-based fluorogenic materials have been utilized by either covalent or noncovalent conjugation of various fluorophores [83]. When a cavitand binds to a specific guest molecule, it can lead to changes in fluorescence, allowing for the development of fluorescent probes [84,85,86]. For instance, Llobet et al. designed a superior performance gas sensor, which consists of quinoxaline-walled thioether-legged cavitand connected to oxygen plasma-treated Au-NP-modified MWCNTs, and which exhibits unprecedentedly high sensitivity in tracing low levels of benzene in dry air, with a wide range of potential applications in environmental monitoring, workplace safety, or medical devices, etc. (Figure 9) [87]. Alternatively, a sensor array can be created by combining supramolecular cavitands and dyes to discriminate subtle differences among similar analytes.

**Figure 10:**
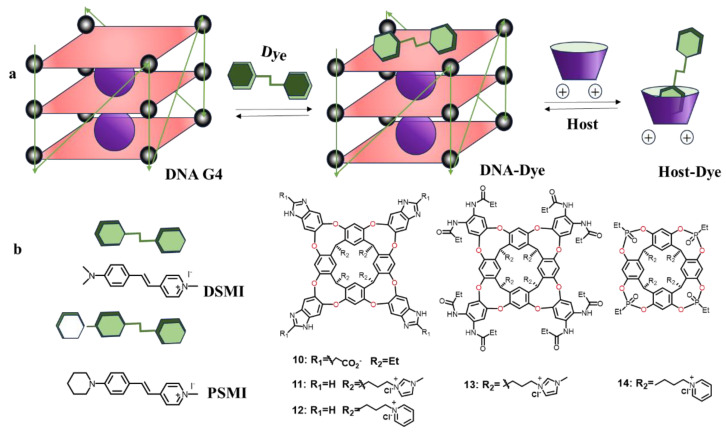
(**a**) “Chemical sniffer” sensing mechanism. (**b**) The chemical structures of cavitands (**10**–**14**) and dyes.

Hooley et al. have demonstrated a “chemical sniffer” for sniffing folded DNA fragments in a unique way (Figure 10) [88]. The sniffing mechanism takes advantage of the fact that the cavitand hosts (**10**–**14**), and dye guests have different affinities for each other and for the target oligonucleotide, resulting in different levels of fluorescence intensity due to the emission of the dye molecules. The sniffer is highly sensitive to the structure of DNA and can differentiate between two types of DNA G4s (those with different structures and folding states). Exposure of eight different structural DNAs to twelve different host–guest fractions was subjected to principal component analysis (PCA), which was shown to fully discriminate the eight different structural strands with a 95% confidence level for all repeated measurements. Similarly, the ability of the sniffer to classify topological types of highly similar G4s sequences was verified by analyzing PCA plots depicting variations in the mean fluorescence and fluorescence values of 23 G4s containing different folding states with twelve different host–guest components. In addition, through biological experiments, they found that the sniffer can also detect variation in the folding pattern of G4s in different types of complex media, mixtures of nucleotides containing interfering small molecules. This straightforward, non-invasive sensing technique utilizes a simple multicomponent approach to ensure broad applicability. They also found that functionalization of the upper and lower rims of the cavitand with different motifs can incorporate different fluorescent dyes, displaying multiple recognition mechanisms that can selectively sense multiple targets such as post-translationally modified peptides, kinase substrates, drugs of abuse, and insect pheromones [89]. For example, the cavitand can bond trimethylammonium (R-NMe^3+^)-tagged guest dyes to form a novel sensor that allows for selective indicator displacement-based sensing of a variety of choline derivatives, and also controls the intracellular localization of bound targets. Incubation of the host–guest complexes with cells did not result in significant cell death, demonstrating favorable biotoxicity.

## 6. Battery Materials

In recent years, lithium-ion batteries (LIBs) have been widely used in various aspects of production and human life due to their high energy density and long cycle life [90,91,92]. Organic ligands, as important components of polyacid-based complexes, play a crucial role in the structure and properties of these complexes. In this regard, calix[4]resorcinarene-based cavitands have a unique cavity structure and easy modification. Therefore, they are widely used to construct coordination compounds with tunable structures and diverse properties. Meanwhile, the calix[4]resorcinarene-based cavitand is favorable for lithium ion migration, which makes its synthesized polyacid-based complexes advantageous in the field of energy storage.

**Figure 11:**
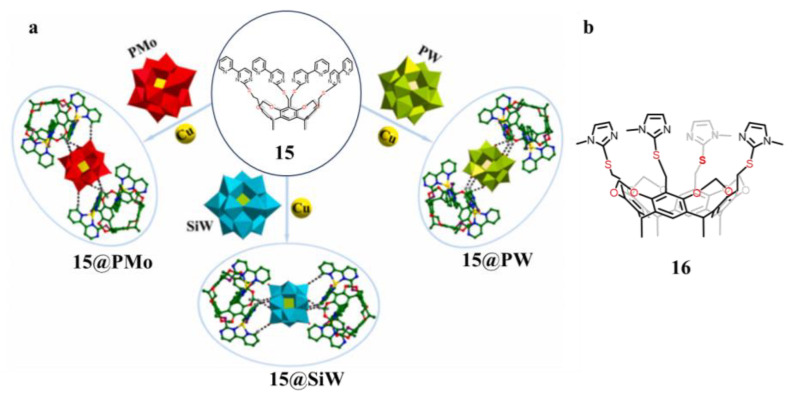
(**a**) Schematic representation of the synthetic strategy for **15@PMo**, **15@SiW** and **15@PW**. (**b**) Structure of kite cavitand (**16**). Reproduced from [93] with permission from the Journal of Alloys and Compounds.

Ma’s group synthesized a series of isostructural polyacid-based calix[4]resorcinarene-based cavitand complexes with PMo, SiW, and PW, respectively, using the cavitand (**15)** as a building unit [93]. Calix[4]resorcinarene organic ligands improved the structural stability of the polyacids in the electrolyte (Figure 11). Meanwhile, the unsaturated N atoms of the organic ligands are more likely to grab Li ions and enhance lithium performance. The composite of polyacid-based complexes (**15@PMo-15@PW**) with graphene oxide (GO) milling improved the conductivity of the material. The ball-milled composite has a smaller size and larger specific surface area, which allows Li^+^ to contact more active sites. Meanwhile, the study of different polyacid oxides revealed that the W=O bonding energy of silico-tungstic acid is larger relative to the W=O/Mo=O bonding energy of phospho-tungstic acid and phosphomolybdic acid, which makes it easier for Li^+^ to react electrochemically with it. In conclusion, thanks to the ability of the uncoordinated N of the cupro-aromatic organic ligand to capture Li^+^, the exposure of more active sites after composite with GO, and the stronger W=O bonding energy in the silico-tungstic states, **15@SiW/GO** exhibits excellent electrochemical performance as an anode material for LIBs.

Ma’s group prepared the complex **16@SiW/GO** by mechanical ball milling of hydrogen-bonded 3D polyacid-based mixture **16@ SiW** with GO, investigated the electrochemical properties of the composite, and further explored the effect of the amorphous state of the polyacid-based complex on its electrochemical properties [94]. Mixture **16@SiW** was obtained by self-assembly of cavitand (**16**), cobalt ions and SiW. GO was partially reduced during the ball milling process, which improved the electrical conductivity of the composites, while some of the **16@SiW** mixture produced an amorphous state, which exposed more active sites and thus promoted the diffusion of Li^+^ in the electrode materials. Among these, the composite **16@SiW/GO** with suitable GO content showed excellent multiplicative performance, and still possessed a high specific capacity of 606 mAh g^−1^ at a current density of 5 Ag^−1^, significantly higher than some previously reported polyacid-based anode materials, which further demonstrated that the amorphous state of polyacid-based complexes improved the electrochemical performance of the materials. This research work provides an experimental basis and scheme for the design and synthesis of polyacid-based LIBs anode materials with high specific capacity performance.

## 7. Drug Delivery

Within the rapidly growing field of host–guest chemistry, disease diagnostic and therapeutic strategies based on supramolecular assemblies are springing up. Cavitands have also attracted attention as possible smart nanocarriers that can selectively escort chemotherapeutic drugs to diseased tissues [95,96,97]. Cavitands can utilize high encapsulation capacity and accommodate hydrophilic and hydrophobic drugs thanks to their specific cavities, and are decorated with different stimuli-responsive functional groups to facilitate controlled drug release [98]. Furthermore, drug encapsulation via supramolecular methods overcomes most chemotherapeutic challenges, such as improving water solubility, bioavailability and stability, and minimizing resistance [99,100,101]. This method provides a straightforward and potentially effective treatment to battle bacterial infections and facilitate selective therapy. As research in this field progresses, further advancements in cavitand-based drug carriers are anticipated, expanding the scope of supramolecular assemblies for innovative and tailored disease diagnostic and therapeutic interventions [102].

**Figure 12:**
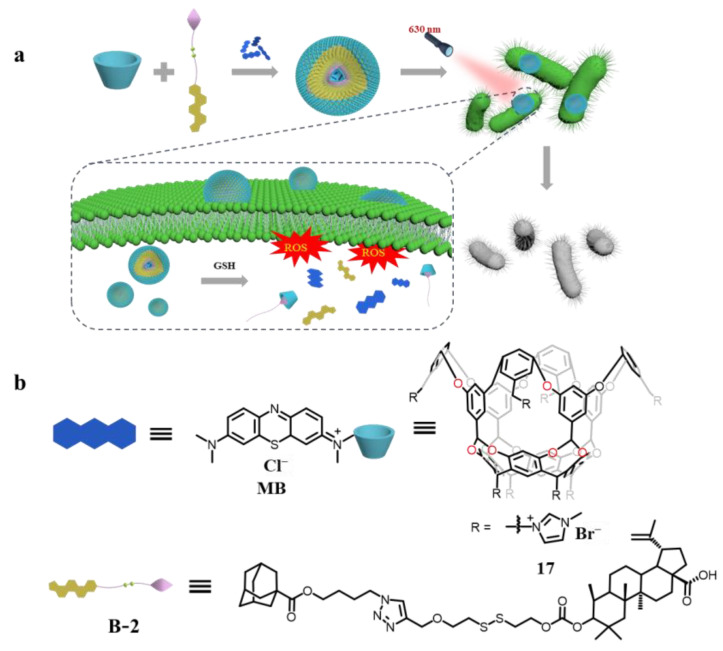
(**a**) Schematic illustration of supramolecular prodrug vesicles. (**b**) Chemical structures of the delivery system. Reproduced from [103] with permission from Chinese Chemical Letters.

Our group presents a chemo-photodynamic antimicrobial strategy based on supramolecular pro-drug vesicles to inhibit bacteria with high concentrations of glutathione (GSH), such as *E. coli* [103] (Figure 12). This system was constructed from a water-soluble cavitand (**17**), a photosensitizer methylene blue (**MB**), and a prodrug guest (**B-2**), which consists of adamantane and betulinic acid (a natural antibiotic) linked by disulfide bonds. Strong host–guest interaction allows the assembly of **17** and guest B-2 into supramolecular prodrug vesicles in aqueous solution. Subsequently, the encapsulation of MB in the cavity of **17⸧B-2** vesicles, and photosensitizer-loaded prodrug beads (**17⸧B-2**@MB) were also successfully prepared. In the meantime, upon excitation at 630 nm, photosensitizers generated ROS, effectively eradicating bacteria through combined chemo-photodynamic therapy. High concentrations of GSH cleave disulfide bonds in the prodrug guest, causing antibiotics and photosensitizers to be released inside the cell. The morphology and size of the assemblies were characterized with a scanning electron microscope (SEM) and the hydrodynamic diameters were determined with dynamic light scattering (DLS). An increase in vesicle particle size after MB encapsulation can be observed, indicating successful photosensitizer encapsulation. The variation in UV-absorbed intensity suggests that the presence of GSH promotes the cleavage of prodrug vesicles, leading to the release of drug and photosensitizer. In order to test the bacteriostatic activity of the vesicles, the morphology of the bacteria was observed using SEM. The morphology of *E. coli* was broken under 630 nm light irradiation.

## 8. Conclusions

In summary, we have given a brief overview of the applications of these cavitands in catalysis, separation and purification, polymeric materials, sensing, battery materials, as well as drug delivery. During the last five decades, the realm of molecular recognition has advanced significantly, aiming to achieve precise and selective identification of intricate small molecules while discerning complex mixtures of compounds. The foundational principles governing non-covalent interactions, which were derived from the examination of synthetic host–guest systems, have greatly enriched our comprehension of molecular recognition phenomena in both chemical and biological contexts. Calix[4]resorcinarene-based cavitands, as a kind of specific concave macrocyclic host, have contributed and evolved tremendously in this regard along with many other macrocycles. It is worth noting that these cavitands may not be tailor-made for some of the aforementioned applications. However, the investigations demonstrated the great potential of cavitand hosts and their applications in other areas, such as gene delivery, are waiting to be unveiled. These applications will definitely inspire the design of more cavitands with novel functionality in the future, enriching the diversity of the vast field of supramolecular chemistry.
